# Regulation of eco-tropic human immunodeficiency virus type-1-infection by sterile alpha motif and histidine-aspartic domain containing protein-1 in a microglial cell line: a novel in vitro model for studying HIV infection and latency in microglia

**DOI:** 10.1007/s13365-025-01280-9

**Published:** 2025-10-21

**Authors:** Brita Ostermeier, Clarissa Halpern, Sanjay B. Maggirwar

**Affiliations:** Microbiology, Immunology, and Tropical Medicine, The George Washington University, 2300 Eye Street NW, Washington, D.C., 20037 USA

**Keywords:** *EcoHIV*, *CHME5 cells*, *SAMHD1*, *HIV latency and reactivation*, *Microglia*

## Abstract

Microglia are considered the main human immunodeficiency virus (HIV) reservoirs in the central nervous system (CNS) due to their ability to become productively infected, produce new infectious HIV virions, and support HIV latency. The anatomical location of microglia necessitates the use of in vitro HIV infection models. However, currently available in vitro models are laced with limitations, including their suboptimal HIV infection rates, poor experimental tractability, and low affordability. Therefore, we sought to develop a new in vitro infection model that addresses these concerns. Here, we confirmed that microglia express sterile alpha motif and histidine-aspartic domain-containing protein-1 (SAMHD1), an antiviral mechanism that opposes HIV replication. We show that administration of simian immunodeficiency virus (SIV)-derived Vpx virus-like particles (VLPs) can reduce the levels of SAMHD1, thus allowing for increased infectivity in EcoHIV-infected CHME5 microglial cell line. With this model, we achieved higher initial rates of HIV infection. We could also track the cells using eGFP expression during active replication, latency, and latency reversal. Further, we developed a CHME5-EcoHIV + cell line using fluorescence-activated cell sorting (FACS). Despite high infectivity of HIV in CHME5 cells, we confirmed that limited latency reversal occurs following their exposure to conventional latency reversing agents (LRAs). Our novel microglia infection model saves researchers time and money and, due to its ease of use, can rapidly contribute to curative research in the field.

**Supplementary Information:**

The online version contains supplementary material available at 10.1007/s13365-025-01280-9.

## Introduction

Human immunodeficiency virus (HIV)-infected immune cells enter the central nervous system (CNS) and disseminate the virus during the acute phase of infection (Valcour et al. 2012). Once within the CNS, HIV infects brain-resident cells such as perivascular macrophages, oligodendrocytes, astrocytes, and microglia, but not neurons (Bagasra et al. 1996; Yadav and Collman 2009; Silvana et al. 2021; Swingler et al. 2023; Woodburn et al.). Microglia are widely recognized as the primary cellular reservoir of HIV in the CNS (Wallet et al. 2019; Donoso et al. 2022; Tang et al. 2023). These long-lived brain-resident immune cells harbor latent, replication-competent HIV (Abreu et al. 2019; Tang et al. 2023), making them a major barrier to curing HIV infection and a key contributor to HIV-associated neurological disorders (HAND). Due to the anatomical location of microglia, the study of viral persistence in microglia is technically challenging. Post-mortem brain samples are terminal and limited; as such, in vitro infection models are essential for uncovering the mechanisms of HIV infection & latency in microglia, thereby developing curative strategies against HIV infection of the CNS.

Currently, there are three major in vitro models available to study HIV infection in human microglia: the C20 and HC69 cell lines *(*Garcia-Mesa et al. 2017a), monocyte-derived microglia (MMG) (Rawat and Spector 2017), and induced pluripotent stem cell-derived microglia (iPSC-MG) (Wang et al. 2023; Boreland et al. 2024). Each of these models has its pros and cons; however, iPSC-MG remains the best in vitro model of the three due to its resemblance to primary microglia and infection efficiency. Specifically, iPSC-MG have microglial gene signatures most similar to primary microglia and evidence of the formation of a virus-containing cellular compartment (VCC) (Rai et al. 2020). Additionally, iPSC-MG have higher HIV-1 p24 release than MMG at high multiplicities of infection (MOIs) (~ 10–15 ng/mL), indicating higher infection rates (Rai et al. 2020).

However, iPSC-MG lack experimental tractability and affordability. HIV infection models in MMG and iPSC-MG utilize wild-type HIV strains such as HIV_Ba−L_, which allows for the study of all stages of infection, but it is more difficult to identify which cells are productively infected without a fluorescent marker. Studies could be performed in these cells by using replication-defective HIV that contains a fluorescent marker; however, this has not yet been reported. Furthermore, methods to generate iPSC-MG are prolonged; for example, iPSCs undergo hematopoiesis (12 days), microglial differentiation (25 days), and maturation (3 days) (Rai et al. 2020). The HIV infection of the iPSC-MG can be monitored for up to an additional 14 days (Rai et al. 2020). It could take almost 2 months (54 days) to use this model of infection once. In addition to the time, expensive supplements, including IL-34, TGFβ−1, M-CSF, CD200, CX3CL1, and specialized iPSC growth media are required for differentiation and maturation. Already differentiated iPSC-MG are commercially available, saving researchers approximately 40 days, although they remain costly, potentially up to thousands of dollars. While iPSC-MG are the most similar to primary microglia, the cost of generating or buying iPSC-MG can be prohibitive to the laboratories. Due to their affordability, MMG and C20/HC69 microglia are more appealing models than iPSC-MG for preliminary experiments. However, both the C20/HC69 and MMG models lack the resemblance to primary microglia (Rai et al. 2020). MMG also lack robust infection rates as mentioned previously (Rai et al. 2020), and HC69 microglia are already infected with HIV, prohibiting the study of the full course of HIV infection.

Thus, there is a need for developing additional in vitro models of HIV infection in microglia that have a robust infection rates, a fluorescent marker for tracking productive infection, latency, and reactivation, and are inexpensive. To this end, we developed a novel microglial HIV infection model: treatment with simian immunodeficiency virus (SIV)-encoded viral protein x (Vpx) virus-like particles (VLP) and EcoHIV NL4-3-eGFP infection in an immortalized microglial cell line of rat origin, CHME5. This model aims to address the concerns of other models by illustrating increased infection efficiencies, experimental tractability through eGFP, and affordability. This model can serve as an inexpensive method for initial hypothesis testing, saving researchers both time and money. While it will still be important to validate any findings in primary or iPSC-MG, we believe that this infection model will be useful for discovery-stage experiments.

## Methods

### **Cell Cultures**

The C20 and HC69 cells (kind gift of Dr. Jonathan Karn, Case Western Reserve University School of Medicine) were cultured in DMEM with low glucose, pyruvate, with no phenol red (#11054020, ThermoFisher) and supplemented with 1% Heat-inactivated FBS, 1X penicillin/streptomycin, and 1X GlutaMAX™ (#35050061, ThermoFisher). Cells were passaged every 2–3 days. HC69 cells were cultured with an additional supplement, 1 µM dexamethasone (#D4902, Sigma Aldrich) (Garcia-Mesa et al. 2017a). The CHME5 microglial cell line (kind gift of Dr. M. Tardieu, University of Medicine Paris-South, France) was cultured in DMEM 5% Heat-inactivated FBS, 1% penicillin/streptomycin, and 1X GlutaMAX™. Cells were passaged every 2–3 days with 0.25% Trypsin-EDTA. To generate an EcoHIV-infected CHME5 cell line, the cells were sorted for eGFP + expression on day 2 post-infection using a Sony SH800Z Cell Sorter. The eGFP + cells were cultured until day 10 and then cryopreserved or used for latency reversal studies.

### **Flow Cytometry**

The C20, HC69, and CHME5 cells were first stained with viability dye Zombie Aqua™ (#423101, Biolegend) and washed with staining buffer (2% Heat-inactivated FBS in 1X PBS). After viability staining, the CHME5 cells were stained with extracellular markers human CD4-PE (SK3, Biolegend) and rat CD4-PE (OX-35, Biolegend) when applicable. After extracellular staining, all cells were fixed with 4% PFA. The cells were then acquired via flow cytometry using a BD LSR X10 Fortessa™ or a BD Celesta™.

### **EcoHIV Virus Generation and Quantification**

The EcoHIV-NL4-3-eGFP plasmid (kind gift of Dr. David Volsky, Icahn School of Medicine at Mount Sinai, New York, NY) was used to transfect Lenti-X™ 293 T cells (#632180, Takara Bio) using FuGENE^®^ HD (#E2311, Promega). After 2–3 days, Retro-Concentin™ (#RV100A-1, System Biosciences) was added to the supernatant at 4 °C overnight to aid in virus concentration. Then, the virus was pelleted at 1500 xg for 30 min at 4 °C. Virus pellets were resuspended in cold PBS, flash frozen, and stored at −80 °C in 50 µL aliquots. EcoHIV p24 was quantified using the Quantikine™ HIV-1 Gag p24 ELISA kit (#DHP240B, R&D Systems^®^).

### **EcoHIV Infection of CHME5**

100,000 CHME5 cells were infected with 400 ng of EcoHIV NL4-3-eGFP. Cells were passaged every 2–3 days for up to 11 days. On days 2, 4, 7, and 10, cells were collected for flow cytometry. On day 10 post-infection, the cells were reactivated by adding the following latency-reversing agents (LRAs): PHA-L (1X = 1 µg/mL) (#43-178-45MG, Fisher Scientific), SAHA (1X = 300 nM) (#SML0061-5MG, Sigma Aldrich), Ingenol (1X = 100 nM) (#50-201-1407, Fisher Scientific), and TNF-α (1X = 10 ng/mL) (#T6674-10UG, Sigma Aldrich) directly to the culture medium. After 24 h, the cells were collected to confirm reactivation via %eGFP + using flow cytometry.

### Immunofluorescent Imaging

For mCAT-1 imaging, 100,000 CHME5 cells were plated on poly-d-lysine treated (Thermo Fisher Scientific, #A3890401) 12 mm glass coverslips (VWR, #89015-724) and incubated at 37 °C for approximately 24 h. Cells were then treated with CellMask Orange (Thermo Fisher, #C10045), fixed with 4% PFA, and incubated at 1:30 ratio of mCAT-1 primary antibody (Proteintech, #14195-1-AP) in 1.5% BSA blocking buffer overnight. The next day, 4 µg/mL of goat anti-rabbit IgG AF488 (Thermo Fisher, #A-11008) and DAPI (Fisher Scientific, #EN62248) were added. For EcoHIV infection, 100,000 CHME5 cells were plated on 0.1% gelatin-coated 12 mm glass coverslips with 400 ng EcoHIV and incubated at 37 °C for 48 h. Then, CHME5 were fixed with 4% PFA and stained with DAPI. All images were acquired on an Olympus IX83 inverted fluorescence microscope.

### **Vpx VLP Generation and Quantification**

Vpx VLPs were generated using co-transfection of Lenti-X™ 293 T cells (#632180, Takara Bio) with plasmids encoding SIV Vpx (pscALPS-SIV-MAC-251 vpx) and Vesicular Stomatitis Virus–encoded G protein (VSVG) using Calcium Phosphate (#K278001, Invitrogen™). pscALPS-SIV-MAC251 vpx was a gift from Jeremy Luban (Addgene plasmid #115810; http://n2t.net/addgene:115810; RRID: Addgene_115810). After 48–72 post-transfection, Vpx VLPs were concentrated from cell supernatant by ultracentrifugation at 25,000 rpm for 2 h at 4 °C. After ultracentrifugation, the supernatant was removed, 50 µL of cold 1X PBS was added to each Vpx VLP pellet, and the tubes were stored, covered with parafilm, at 4 °C overnight. The next day, the pellets were combined, aliquoted at 50 µL per tube, and stored at −80 °C.

To quantify the Vpx VLPs, one tube of Vpx VLPs was thawed on ice, 1X RIPA buffer (#89901, Thermo Scientific™) was added at a 1:1 ratio to the tube, and 1 µL of Halt™ Protease and Phosphatase Inhibitor (#78440, Thermo Scientific™) per 100 µL of the sample was added. The diluted Vpx VLP total protein was quantified via a Pierce™ BCA Protein Assay Kit (#A65453, Thermo Scientific™). Using the concentration determined from the BCA assay, 20 µg of the sample was run on a 4–20% SDS-PAGE gel and transferred to a wet PVDF western blot membrane. The blot was stained for total protein with SimplyBlue™ SafeStain (#LC6060, Invitrogen) and quantified using a LICOR Odyssey and FluorChem R imager (Biotechne). The same western blot was washed and stained with a Vpx-specific polyclonal primary antibody (1:1,000, #PA5-144354, Thermo Scientific™) and secondary antibody (#926-32211, LICORBio). The blot was quantified using a LiCor Odyssey and Image Studio (v.5.2, LICORBio) software.

### **Confirmation of Vpx-induced SAMHD1 Degradation**

100,000 CHME5 cells were treated with 220 µg of Vpx VLPs for 4 h. Confirmation of SAMHD1 degradation and viability was determined by flow cytometry. Briefly, the cells were stained with viability dye Zombie Aqua™ (#423101, Biolegend) and washed with staining buffer (2% Heat-inactivated FBS in 1X PBS). For intranuclear staining, samples were permeabilized using eBioscience™ Foxp3 Fixation/Permeabilization Buffer (#00-5523-00, Invitrogen™), washed with BD Perm/Wash™ Buffer (#554723, BD Biosciences), and stained with either SAMHD1-AF647 (#NBP2-73979AF647, Novus Biologicals) or SAMHD1-CoraLite^®^ 594 (#CL59412586100UL, ThermoFisher). Samples were run on a BD LSR X10 Fortessa or a BD Celesta.

### **Vpx VLP Treatment of CHME5**

For Vpx VLP-treated conditions, 100,000 CHME5 were pre-treated with 220 µg of Vpx VLPs 4 h before EcoHIV infection.

### **Statistics**

For experiments with one comparison, single Mann-Whitney tests were used (GP: *=0.0332, **=0.0021, ***=0.0002, ****=<0.0001). For experiments with multiple conditions, multiple Mann-Whitney tests were used (Holm-Šidák correction method, alpha = 0.05) (GP: *=0.0332, **=0.0021, ***=0.0002, ****=<0.0001).

## Results

### CHME5 cell line of rat origin can be infected with EcoHIV

**Fig. 1:**
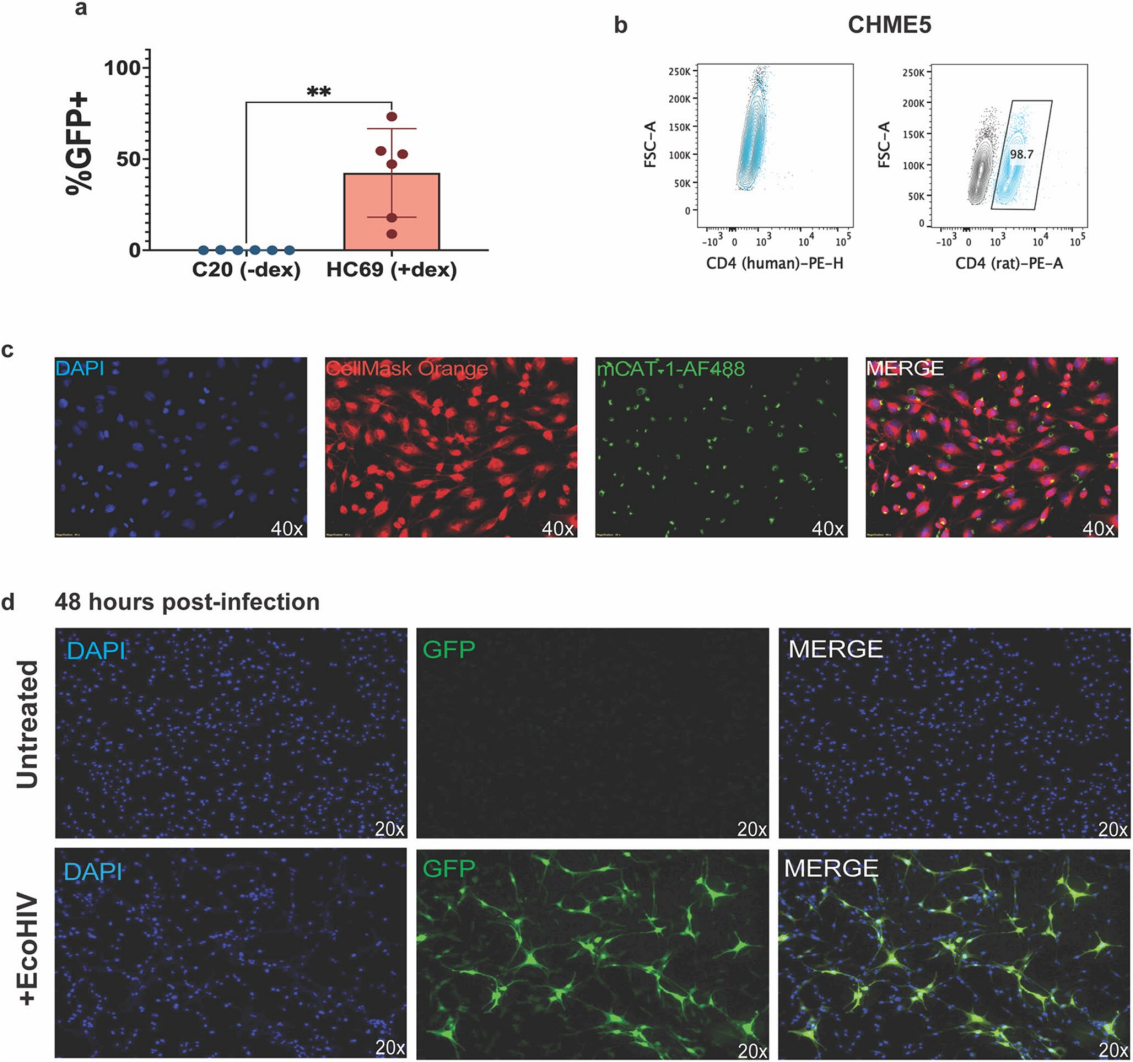
CHME5 rat microglial cells can be infected with EcoHIV. Human C20 and HC69 cell lines, as well as the CHME5 cell line, were cultured under normal culture conditions. The cells were passaged and stained via flow cytometry. Under normal culture conditions with 1 μM dexamethasone, the %eGFP+ HC69 cells was determined via flow cytometry. CHME5 cells were imaged via immunofluorescent microscopy for mCAT-1 expression and EcoHIV infection. a Spontaneous reactivation of HIV in cultures of HC69 (n=6) compared to uninfected C20 microglial cell lines. Mann-Whitney test (*=0.0332, **=0.0021, ***=0.0002, ****<0.0001). b Flow cytometry staining for human-specific and rat-specific CD4 on the CHME5 microglial cell line. c CHME5 expression of EcoHIV receptor, mCAT-1 (fluorescent microscopy). d 48-hours post- EcoHIV NL4-3-eGFP infection of CHME5 (fluorescent microscopy)

The human C20/HC69 HIV infection model in microglia is a less expensive alternative to iPSC-derived microglia or monocyte-derived microglia (MMG) (Garcia-Mesa et al. 2017b). However, the concerns about similarity to primary microglia (Rai et al. 2020) and the applicability for study of the full course of HIV infection remain. Furthermore, we found that the HC69 microglia, which are latently infected with HIV, exhibit spontaneous reactivation of the HIV provirus, despite the presence of dexamethasone (Fig. 1a). Given these concerns, there is a need for a better in vitro infection model that is still affordable for preliminary studies. Since a cell line is one of the more affordable methods, we propose that the CHME5 microglial cell line can fill this need.

CHME5 microglia are reported to be either of rat (Garcia-Mesa et al. 2017a; Figueroa-Hall et al. 2017) or human origin (Vergara et al. 2019; Lisi et al. 2022). We found that our stock of CHME5 is likely of rat origin as only the rat-tropic CD4 clone of antibodies binds to CHME5 microglia (Fig. 1b). Given the rat origin of our CHME5, we sought to infect the cells with eco-tropic HIV (EcoHIV), which utilizes the murine leukemia virus-1 (MuLV-1) envelope protein gp80 for host cell entry (Potash et al. 2005; He et al. 2014). First, we confirmed the expression of EcoHIV receptor, mCAT-1 (Fig. 1c). Next, we established that EcoHIV can infect CHME5 cells, with infection rates at 13% for one representative field quantified via the %eGFP + cells on day 2 post-infection (p.i.) (Fig. 1d).

### SIV Vpx VLPs reduce SAMHD1 levels in CHME5

**Fig. 2:**
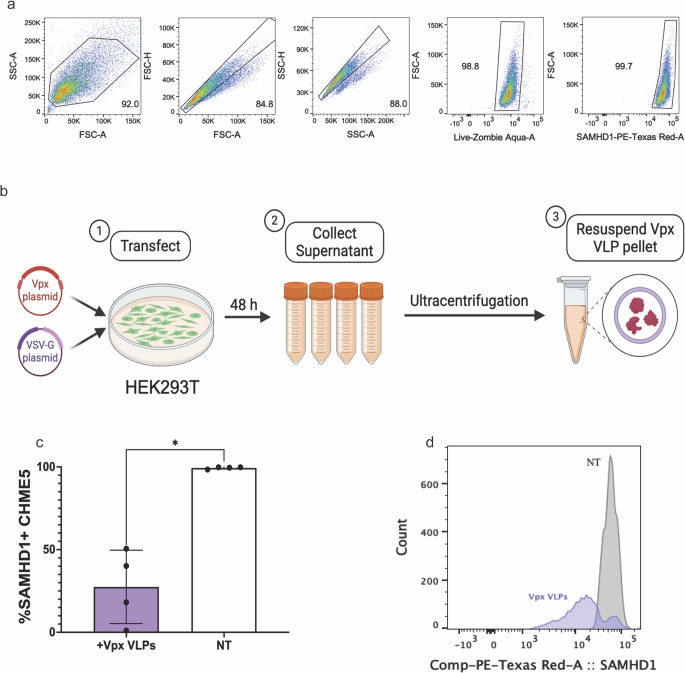
CHME5 microglial cells highly express SAMHD1, which can be reduced via Vpx VLPs. The CHME5 cell line was cultured under normal culture conditions to determine the basal SAMHD1 levels via flow cytometry. HEK293T cells were transfected with both Vpx and VSV-G plasmids to produce Vpx VLPs. CHME5 were treated with Vpx VLPs for 4 h, and SAMHD1 levels were quantified via flow. **a** Gating strategy for microglia through FSC-A vs. SSC-A, single cells FSC-A vs. FSC-H, single cells SSC-A vs. SSC-H, Live cells, and SAMHD1 + cells. **b** Vpx VLP generation methodology (created with BioRender.com). **c** Vpx VLP-treated SAMHD1 expression in CHME5 (*n* = 4) (flow, %). Mann-Whitney test (p values, *=0.0332, **=0.0021, ***=0.0002, ****<0.0001). **d** Representative distribution of SAMHD1 levels in CHME5 cells after 4 h of Vpx VLP treatment, or left without treatment (NT)

To further improve the EcoHIV infection model in CHME5, we next sought ways to increase infectivity. CHME5 cells, like other myeloid cells, highly express SAMHD1 (Fig. 2a), which contributes to suboptimal in vitro HIV infection rates in myeloid cells (Goujon et al. 2006; Hrecka et al. 2011; Laguette et al. 2011; Sunseri et al. 2011; Hofmann et al. 2012; Rai et al. 2020; Akiyama et al. 2021). Vpx VLPs enhance infection rates in myeloid cells by targeting SAMHD1 for degradation (Goujon et al. 2006; Hrecka et al. 2011; Laguette et al. 2011; Akiyama et al. 2021). As such, to enhance infection rates, Vpx VLPs were generated by co-transfecting HEK293T cells with plasmids encoding for SIV-derived Vpx and VSV-G (Fig. 2b), quantified using a whole protein blot and a SAMHD1-specific western blot to determine the average percentage of Vpx protein in the Vpx VLP aliquots (Supplementary Fig. 1, Supplementary Table 1), and administered into the CHME5 cultures. Vpx VLP treatment of CHME5, for 4 h, significantly reduced the SAMHD1 levels to an average of 27.44% (SD 22.18) (*p* = 0.029) (Fig. 2c, d). However, while Vpx VLP treatment reduces SAMHD1, it also appears to reduce the viability of the cells (*p* = 0.029) (Fig. 2e).

### Enhanced infectivity of EcoHIV in CHME5 following Vpx VLP treatment

**Fig. 3:**
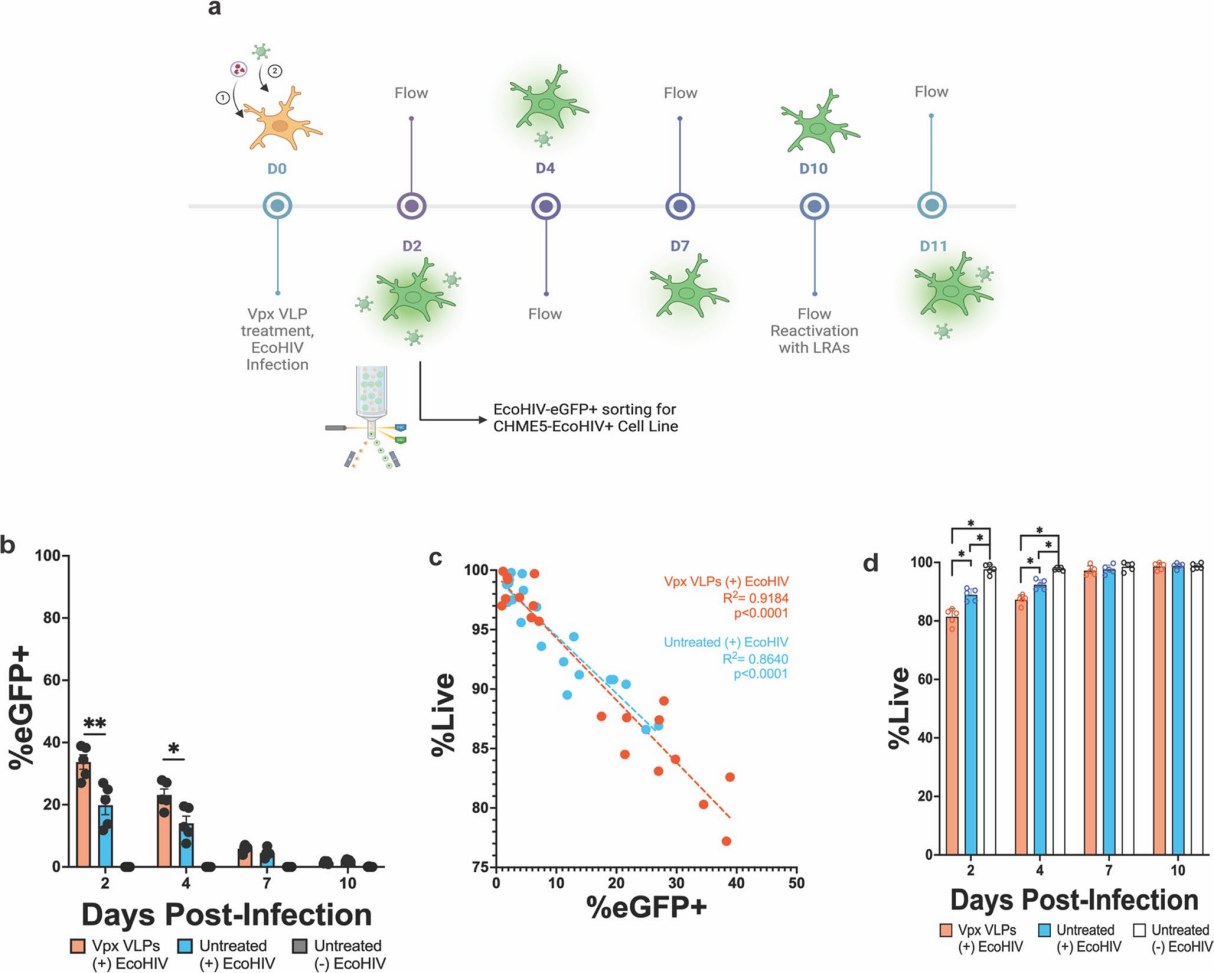
SIV-derived Vpx virus-like particle (VLP) administration increases EcoHIV infection rates in CHME5 microglia. The CHME5 cell Line was either pre-treated or not treated with Vpx VLPs for 4 h prior to EcoHIV NL4.3-eGFP infection. EcoHIV infection of CHME5 was monitored via imaging and flow cytometry on day 2 post-infection (p.i.) and followed through day 10 p.i. Viability was also monitored via flow cytometry in these cells collected from days 2–10 p.i. **a** Infection model (created with BioRender.com). **b** EcoHIV infection curve in CHME5 cells following their exposure to Vpx VLPs (*n* = 5) (flow, %). **c** Viability vs. GFP+ (flow, %). Vpx VLPs (+) EcoHIV (*n* = 20, orange) and Untreated (+) EcoHIV (*n* = 20, blue) conditions were compiled from days 2–10 and analyzed separately via a simple linear regression. **d** CHME5 viability on days 2–10 after Vpx VLP treatment and EcoHIV infection (*n* = 5) (flow, %). Mann-Whitney test (p values, *=0.0332, **=0.0021, ***=0.0002, ****<0.0001)

To test whether Vpx VLP targeting of SAMHD1 could enhance HIV infection in microglia, we developed a novel model of infection (Fig. 3a). When pre-treated with Vpx VLPs, EcoHIV infection rates on day 2 were significantly enhanced from an average of 19.82% (SD 6.73) to an average of 33.70% (SD 5.22) (*p* = 0.0159) (Fig. 3c). This level of %eGFP + cells remain sustained on day 4 p.i. (*p* = 0.0317) (Fig. 3c). The %eGFP + cells follow an infection curve as EcoHIV enters latency (Fig. 3c). There is a significant negative correlation between %eGFP and viability (R^2^ = 0.9101) (*p* < 0.0001) (Fig. 3d). Additionally, Vpx VLP-treated and EcoHIV-infected CHME5 have significantly lower viability than EcoHIV-infected or untreated CHME5 on day 2 (*p* = 0.0079, *p* = 0.0079) and day 4 p.i. (*p* = 0.0079, *p* = 0.0079) (Fig. 3e). Flow gating strategies are exhibited in Supplementary Fig. 2.

### EcoHIV latency reversal

**Fig. 4:**
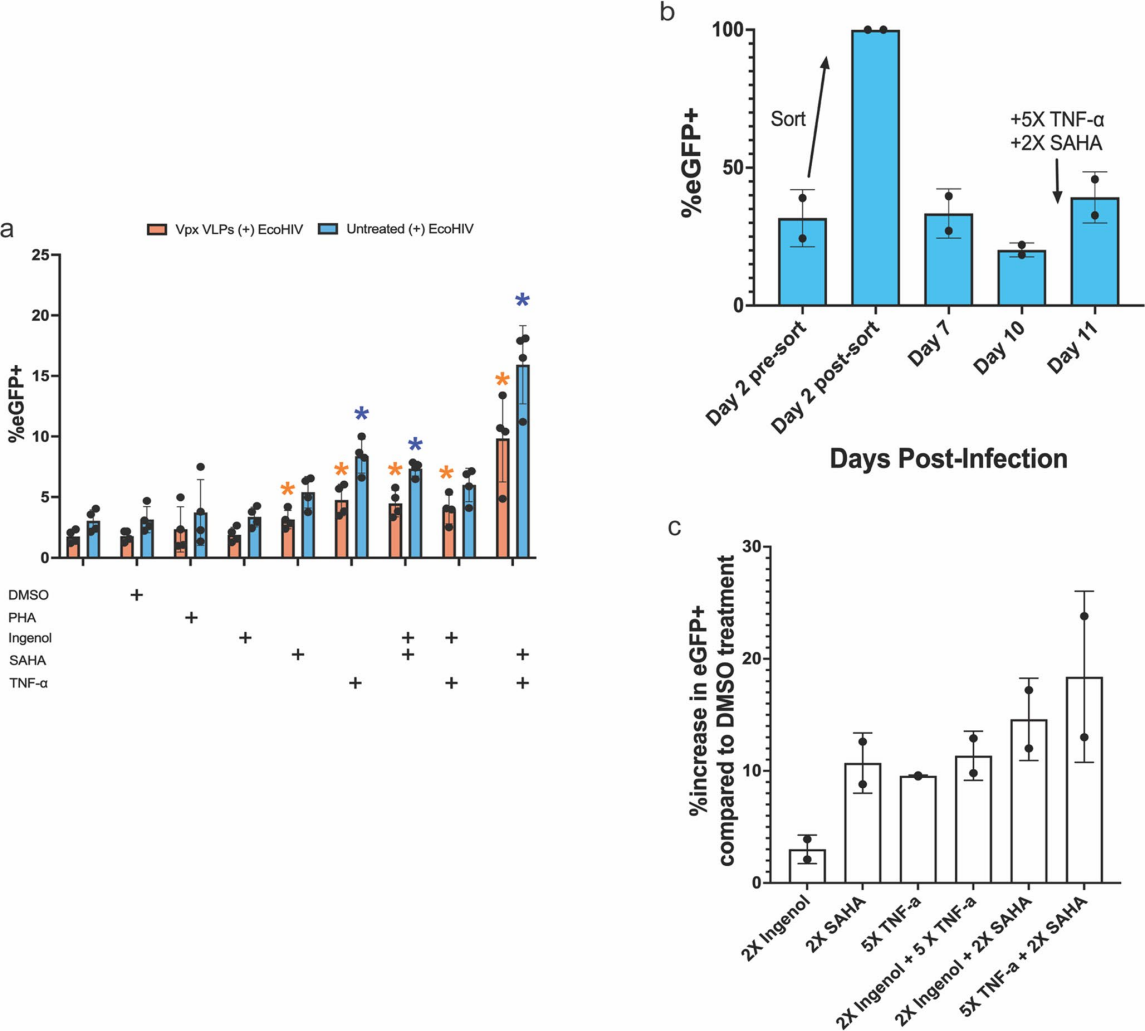
EcoHIV-infected CHME5 exhibits latency reversal. The CHME5 cell line was either pre-treated or not with Vpx VLPs for 4 h prior to EcoHIV-eGFP infection. EcoHIV infection of CHME5 was monitored via imaging and flow cytometry on day 2 post-infection (p.i.) and followed through day 10 p.i. After day 10 p.i., LRAs were added to the cells to induce latency reversal. Latency reversal was quantified via the %eGFP + cells using flow cytometry after 24 h. Some EcoHIV-infected CHME5 cells were sorted for the eGFP + population to create an EcoHIV-infected CHME5 sub-line. The CHME5-EcoHIV-eGFP + cells were followed through day 10 p.i. and reactivated with LRAs. **a** 24-hour reactivation with LRAs (*n* = 4) (flow, %). **b** CHME5-EcoHIV-eGFP + sorted curve on days 2–11 (*n* = 2) (flow, %). **c** 24-hour reactivation of CHME5-EcoHIV-eGFP + sorted curve with multiple LRAs (*n* = 2) (flow, %). Mann-Whitney test (p values, *=0.0332, **=0.0021, ***=0.0002, ****<0.0001)

To confirm EcoHIV latency, infected cells were treated with a range of latency-reversing agents (LRAs), primarily those that have proven activity in HIV-infected T cells (Spivak et al. 2015; Venkatachari et al. 2015; Passaes et al. 2017), on day 10 p.i., and were acquired by flow cytometry after 24 h. The singular LRAs, such as PHA, Ingenol, and SAHA, failed to reactivate EcoHIV as quantified by eGFP + expression, whereas 5X TNF-α treatment led to eGFP expression up to an average of 15.93% (SD 3.23; *p* = 0.029) (Fig. 4a). It appears that these LRAs are not effective in microglial cells at the concentrations that are known to reactivate latent HIV in T cells (Spivak et al. 2015; Venkatachari et al. 2015; Passaes et al. 2017). TNF-α – a key inflammatory cytokine involved in the brain’s response to HIV-infection – is known to stimulate HIV replication in microglia (Wilt et al. 1995). Thus, it is conceivable that TNF-α alone, albeit at higher concentrations, may have the ability to reverse the HIV latency in microglia. Based on these results, we propose that the high concentrations of TNF-α, that is often observed in the vicinity of activated cells in the CNS (Pan et al. 1997; Rajora et al. 1997; Gonzalez Caldito 2023), are adequate to reactivate latent HIV in microglia. Similarly, for the Vpx VLP-treated/untreated and EcoHIV-infected CHME5 cells, 2X SAHA (*p* = 0.029), 2X Ingenol + 5X TNF-α (*p* = 0.029), 2X Ingenol + 2X SAHA (*p* = 0.029), and 5X TNF-α + 2X SAHA (*p* = 0.029) all significantly reactivated EcoHIV compared to a DMSO control (Fig. 4a).

We performed additional experiments to better understand the latency reversal of HIV in microglial cells. EcoHIV-infected CHME5 cells, day 2 p.i., were sorted to clonally select a cell Line population with 100% EcoHIV infection (Figs. 3a and 4b). After most of these cells entered HIV latency, the cells were treated with the various combinations of LRAs that are outlined above. We found that the indicated LRAs reactivated up to an average of 18.40% (SD 7.64) (% increase from DMSO control) of infected cells, with 5X TNF-α and 2X SAHA being the most potent LRAs (Fig. 4b, c). The reactivation rate of the sorted CHME5 cell line appears to be similar to the non-sorted cells, indicating that despite a higher percentage of infected cells post-sort, there is a limit to reactivation from latency in microglia.

## Discussion

Current in vitro models of HIV are lacking in terms of affordability, tractability, and infection efficiencies. To fill this gap in research, we developed a novel HIV infection model in microglia in which the CHME5 microglial cell line is pre-treated with Vpx VLPs and infected with EcoHIV NL4-3-eGFP. Our infection model alleviates previous concerns and provides a new method for studying the full course of HIV infection and latency in microglia for the study of curative treatment modalities in vitro.

Complementing previously published data on the rat origin of CHME5 (Garcia-Mesa et al. 2017a; Figueroa-Hall et al. 2017), we confirm the rat origin of our CHME5 cell line by rat-specific CD4 staining of CHME5 cells compared to staining with human-specific CD4 clone. For the purpose of developing an infection model, we confirmed the expression of murine leukemia virus type-1 (MuLV-1) receptor mCAT-1/ERR/Rec-1 in CHME5 cells. In the EcoHIV NL4-3-eGFP viral construct, the coding region of gp120 in HIV-1/NL4-3 was replaced with that of gp80 from ecotropic MuLV-1, a retrovirus that infects rodent cells. EcoHIV NL4-3-eGFP does not use CD4, CXCR4, CCR5 receptors that are conventionally required for HIV infection, or other viral receptors such as DC-SIGN or CLEC-2 for entry into cells and is highly specific to cells expressing mCAT-1 (Alfar et al. 2024). The resulting chimeric virus construct, EcoHIV, productively infects murine lymphocytes, but not human lymphocytes (Potash et al. 2005; He et al. 2014).

Due to its ecotropism, conducting EcoHIV infection of cells in vitro is also safer to the researchers, only requiring measures equivalent to basic biosafety level-2 (BSL-2), while HIV can require advanced BSL-2 + containment. EcoHIV infection models in mice support viral replication in CD4 + lymphocytes, macrophages, and microglia (Hadas et al. 2007; Kelschenbach et al. 2012; He et al. 2014). However, in the infected rodent brain, microglia are the predominant cell type harboring EcoHIV, both in vitro and in vivo, with prominent HIV-1 mRNA expression within 24 h in culture and seven days post-infection in live rodents (Li et al. 2020, 2021, 2023). Additionally, in vivo EcoHIV infection models in mice and rats, while not the same as HIV-1 infection, can replicate many aspects of HIV-associated neurocognitive disorders (HAND) (Li et al. 2020, 2021, 2023). This further supports the biological relevance of EcoHIV in an in vitro infection model of microglia. EcoHIV is also enhanced by inserting eGFP upstream of the intact *nef* gene, allowing for the visualization of active EcoHIV replication and latency (He et al. 2014; Kim et al. 2024). EcoHIV has been utilized extensively for in vivo mouse and rat studies, however, in vitro models, particularly for the CNS reservoir, are important for foundational research. EcoHIV infection rates in rat CHME5 microglia exhibit an infection rate of approximately 13%. While this infection rate is still useful, we sought to further increase the infection rate in the CHME5 to have a much larger infected and latent population.

Interestingly, similar to a previous study of HIV_Ba−L_ infection in iPSC-MG (Rai et al. 2020), we find that HIV infection in microglia appears to be related to cell death. In our model, there is a significant negative correlation between the %eGFP + cells and the viability of the cells (*p* < 0.0001). The higher the levels of replicating EcoHIV, the lower the viability. So, we predict that the decreased viability in the Vpx VLP-treated and EcoHIV-infected CHME5 cells may be primarily due to the combination of reduction in SAMHD1 and increased EcoHIV infection rates. Myeloid cells tend to have more resistance to apoptosis than CD4 T cells. In the latently infected U937 monocytic cell line, the apoptotic pathway is blocked through the inhibition of pro-caspase-3 (Tanaka et al. 1999). If myeloid cells and microglia resist apoptosis, “shock and kill” cure strategies may be difficult to implement. With our infection model, researchers can study cell death mechanisms in microglia, which will inform future “shock and kill” strategies.

To increase EcoHIV infection in CHME5, we first sought to target SAMHD1. SAMHD1 is known to contribute to sub-optimal in vitro HIV infection rates in myeloid cells (Goujon et al. 2006; Hrecka et al. 2011; Laguette et al. 2011; Sunseri et al. 2011; Hofmann et al. 2012; Rai et al. 2020; Akiyama et al. 2021) by hydrolyzing deoxynucleoside triphosphates (dNTPs), thereby lowering the levels of dNTPs in the cells (Lahouassa et al. 2012). These low levels of dNTPs hinder the process of reverse transcription, which HIV needs to produce new virions. Myeloid cells have been reported to have high levels of SAMHD1, such as primary microglia, iPSC-MG, and MMG (Rai et al. 2020) while C20 and HMC3 microglial cell lines have limited expression of SAMHD1 (Rai et al. 2020). HIV-1 infectivity in myeloid cells in vitro is likely opposed by these high levels of SAMHD1, making the study of HIV infection in these cells difficult. To increase infection rates in in vitro myeloid cell models and counteract the anti-viral mechanisms of SAMHD1, researchers are using Vpx. Vpx is an SIV- and HIV-2-derived accessory protein, which targets SAMHD1 for degradation in the nucleus (Hofmann et al. 2012). HIV-1 does not encode for Vpx and thus remains vulnerable to SAMHD1-mediated restriction. When HIV-1 is altered to package SIV-derived Vpx, HIV is more infectious in monocyte-derived macrophages and monocyte-derived dendritic cells (Sunseri et al. 2011; Mahboubi et al. 2018). Another way to deliver Vpx to cells is through Vpx-containing VLPs (Vpx VLPs), which are generated by Vpx and VSV-G plasmid co-transfections in HEK293T cells. Vpx VLPs enhance HIV-1 infection rates in both macrophages and dendritic cells (Goujon et al. 2006; Hrecka et al. 2011; Laguette et al. 2011). Vpx VLPs also enhance HIV-1 infection as measured by p24 production in microglia through an MMG model (Akiyama et al. 2021). Indeed, we find that CHME5 cells highly express SAMHD1, which can be reduced with Vpx VLP treatment. However, it is important to note that the viability of the cells decreases after Vpx VLP treatment, indicating that SAMHD1 may be important for microglial cell survival. Our observations are consistent with the previous reports that defined SAMHD1 as a key regulator of cell survival, acting through genome maintenance, cell cycle control, and immune modulation. Its loss or dysfunction can lead to increased DNA damage, altered cell proliferation, and heightened immune activation, impacting cancer progression and immune responses (Clifford et al. 2014; Bonifati et al. 2016; Coquel et al. 2018; Park et al. 2021; Felip et al. 2022; Shao et al. 2024). We believe that the loss of cellular viability in Vpx VLP-treated CHME5 cells is due to the reduction in SAMHD1 levels, and not vice versa.

When we pre-treat CHME5 cells with Vpx VLPs, the number of infected cells was enhanced. So, similar to MMG, SAMHD1 hinders HIV infectivity in CHME5 microglia (Akiyama et al. 2021). Vpx VLP-mediated increases in infection rates surpass infection rates previously reported in iPSC-MG (Akiyama et al. 2021), supporting the use of this model when a larger infected/latent microglial population is needed.

Without any additional treatments or antiretrovirals, EcoHIV starts to enter latency, as is the case in many other HIV host cells. LRA-mediated reactivation of EcoHIV is most potent with the combination of TNF-α and SAHA, yielding high reactivation rate for EcoHIV-infected CHME5. Interestingly, there does appear to be a trend of increased reactivation in the untreated (+) EcoHIV-infected CHEM5 cells compared to the Vpx VLP-treated, however, given the lack of statistical significance, we believe that higher infection rates at the beginning of infection outweigh any slight lowering of reactivation rates. The concentration of LRAs needed to reactivate EcoHIV-infected CHME5 is much higher than the concentration of LRAs used for T-cell infection models (Sarabia et al. 2021). Even when the EcoHIV-infected CHME5 cells are sorted to a 100% eGFP + cell line, latency reversal is limited. Given the difficulty in reactivating EcoHIV in this infection model, careful consideration is needed for future HIV cure strategies aimed at the viral reservoirs in the CNS. For “shock and kill”, not only do the LRAs need to be able to cross the blood-brain-barrier (BBB) and initiate latency reversal safely, but LRA concentrations that may work for T cell latency reversal might be ineffective for microglial cells. This could indicate the need for other combination therapies for the complete elimination of all reservoirs. Additionally, perhaps a “block and lock” (Vansant et al. 2020), or gene therapy (Khamaikawin et al. 2024) approach may better target HIV-infected microglia. We anticipate that the high infection rates, the natural progression into latency, and the ability to reactivate EcoHIV will be useful for researchers who need a higher degree of infectivity in microglial cells for their studies of latency and reactivation.

While we believe that this in vitro model of infection will be a useful tool for the field, there are some limitations to this model. CHME5 are immortalized microglial cells whose origin is controversial and cannot fully model aspects of primary human microglia. CHME5 microglial cells were originally thought to be of human origin, but in 2017, two studies claimed that CHME5 are of rat origin, likely due to early contamination of the original cell lines (Garcia-Mesa et al. 2017a; Figueroa-Hall et al. 2017). One of the studies determined that the contaminated CHME-5 cells are microglia and not astrocytes by measuring glial fibrillary astrocytic protein (GFAP) (Figueroa-Hall et al. 2017). Researchers confirmed the rat origin of these cells by using primers specific for human CYCT1 and rat CYCT1 (Garcia-Mesa et al. 2017a). Despite the rat origin of some CHME5 lines, they exhibit similar TLR-4 signaling responses that are comparable to primary human microglia, indicating that they can be useful in understanding human microglia (Figueroa-Hall et al. 2017). Some recent studies have obtained CHME5 directly from the laboratory of Dr. M. Tardieu and confirmed the cells’ human origin (Vergara et al. 2019; Lisi et al. 2022). Thus, careful consideration should be given before claiming that the infection rates in CHME5 mimic those of human microglia. While the in vitro infectivity rates of EcoHIV in CHME5 cells may not resemble that of HIV infection of human microglia in vivo, the increased infection rates and the natural progression into latency without ART set this model apart from current in vitro human microglial infection models.

The second limitation of this model is that CHME5 is an immortalized cell line. As such, the cell division rates are much higher than those of MMG, iPSC-MG, or primary human microglia. This may be why we see cell death early on (days 2–4) during EcoHIV infection, whereas cell death is noted after day 8 of infection in iPSC-MG (Rai et al. 2020). While the course of infection is accelerated in the CHME5 model, this makes the model appealing, with infection, latency, and reversal taking only 11 days. This model of infection will be useful for early investigations into revealing mechanisms of HIV infection and latency in microglia due to the CHME5 model’s short timeline. iPSC-MG or primary human microglia will continue to be necessary for the validation of the relevance of key findings to HIV-infected human microglia.

## Supplementary Information

Below is the link to the electronic supplementary material.

Supplementary Material 1 (PDF 913 KB)

## Data Availability

Data is provided within the manuscript or supplementary files. Original data files are available upon request.
