# Supplementary material for: Regulation of eco-tropic human immunodeficiency virus type-1-infection by sterile alpha motif and histidine-aspartic domain containing protein-1 in a microglial cell line: a novel in vitro model for studying HIV infection and latency in microglia
Source: J Neurovirol. 2025 Oct 21;31(6):507–18. doi: 10.1007/s13365-025-01280-9 (PMC12701028; doi:10.1007/s13365-025-01280-9)
Supplement: Supplementary file 1 — Supplementary Material 1 (PDF 913 KB) [file 13365_2025_1280_MOESM1_ESM.pdf]

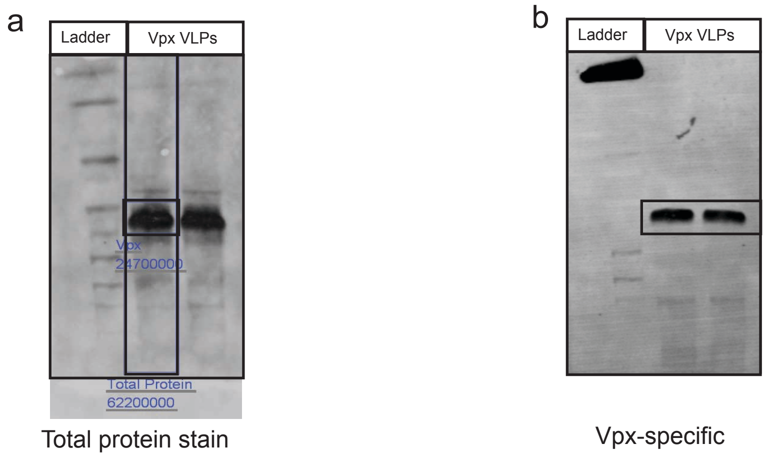

Supplementary Figure 1
Vpx VLP Quantification. Lenti-XTM 293T cells were co-transfected with SIV Vpx and VSVG plasmids to generate Vpx virus-like particles (VLPs). Vpx VLP total protein was quantified by a BCA assay. Then, 20 µg of sample was run on a western blot (WB). The Vpx-specific signal on the WB was divided by the total protein signal on the western blot to determine the %Vpx of total protein. This percentage was used to calculate the concentration of Vpx in the Vpx VLPs after conducting BCA assays. (a) Total protein western blot (b) Vpx-specific (1:1000) western blot

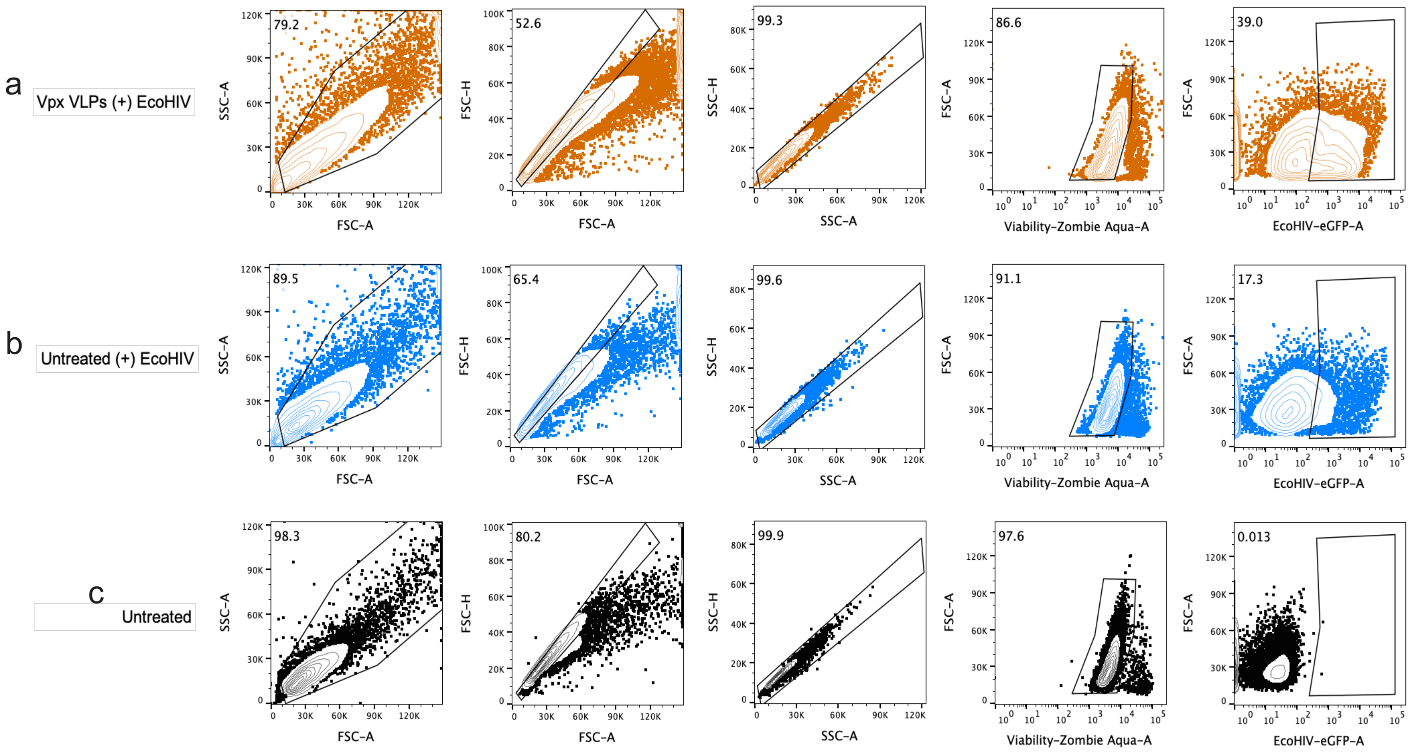

Supplementary Figure 2
Flow Cytometry Gating Strategy for EcoHIV Infection Model In CHME5 Microglia. The CHME5 cell line was either pre-treated or not treated with SIV-derived Vpx virus-like particles (VLPs) for 4 hours prior to EcoHIV NL4.3-eGFP infection. EcoHIV infection of CHME5 was monitored via imaging and flow cytometry on day 2 post-infection (p.i.) and followed through day 10 p.i. Viability was also monitored via flow cytometry in these cells collected from days 2-10 p.i. (a, b, c) Vpx VLP (+) EcoHIV (orange), Untreated (+) EcoHIV (blue), and Untreated (black) example gating strategies.

	BCA Assay Total Protein	Total Protein (mg/mL)/tube	Total Protein Signal WB (20 µg)	Vpx Signal Total Protein WB	%Vpx of Total Protein
**Vpx VLPs**	**1.55**	**0.775**	**62,193,984**	**24,748,160**	**39.79%**

Supplementary Table 1

Vpx VLP Quantification. Lenti-XTM 293T cells were co-transfected with SIV Vpx and VSVG plasmids to generate Vpx virus-like particles (VLPs). Vpx VLP total protein was quantified by a BCA assay. Then, 20 µg of sample was run on a western blot (WB). The Vpx-specific signal on the WB was divided by the total protein signal on the western blot to determine the %Vpx of total protein. This percentage was used to calculate the concentration of Vpx in the Vpx VLPs after conducting BCA assays.
